# Intraoperative pump thrombosis (PT) in HeartMate (HM) III

**DOI:** 10.1111/jocs.16941

**Published:** 2022-09-18

**Authors:** Sathappan Karuppiah, Sudharsan Setty, Ranjit John, Monica Lupei

**Affiliations:** ^1^ Department of Anesthesiology University of Minnesota Medical Center Minneapolis Minnesota USA; ^2^ Department of Surgery, Division of Cardiothoracic Surgery University of Minnesota Medical Center Minneapolis Minnesota USA

**Keywords:** HeartMate III, LVAD, pump thrombosis, transesophageal echocardiogram

## Abstract

Pump thrombosis (PT) is a rare and infrequent complication of HeartMate III left ventricular assist device (LVAD). While there are reports of PT in the postoperative period, to our knowledge, there have been no prior reports on PT in the intraoperative period. Here we present a case of a 24‐year‐old female who required HeartMate III LVAD implantation for progressive heart failure and the intraoperative period was complicated with PT. Managing PT in the intraoperative period is challenging and time‐sensitive because of its rare occurrence and paucity of recommendations in diagnosing the PT.

## 1 INTRODUCTION

The left ventricular assist device (LVAD), a food and drug administration approved mechanical circulatory device for end‐stage heart failure, has brought a significant improvement in patients' survival and quality of life.^1^ Minimizing LVAD related complications is essential for increasing the duration of support on LVAD.^2^ Pump thrombosis (PT) is a dreaded short and long term complication of LVADs. PT refers to the clot formation in any of the pump components including inflow cannula, outflow cannula, or the pump itself.^2^, ^3^ The incidence of PT is about 3%−12% with older generation continuous‐flow LVAD.^3^ However, PT is an uncommon complication of HeartMate (HM) III, the newer generation LVAD and in the intraoperative period. This might be due to better design of the HM III pump that allows for improved washing, preventing clot formation, and deposition and subsequent hemolysis.^4^ We present a case of intraoperative PT with HM III, which required emergent replacement of the LVAD.

## 2 CASE DESCRIPTION

A 24‐year‐old female with a past medical history of postpartum cardiomyopathy, congestive heart failure (CHF) underwent HM II LVAD implantation. Despite being on anticoagulation, the patient suffered an embolic cerebrovascular accident, and the left ventricle was also showing signs of recovery, necessitating the removal of the LVAD. Subsequently, the patient was hospitalized with CHF exacerbation and planned HM III LVAD implantation. She was on dobutamine 2.5 mcg/kg/min preoperatively to manage her CHF before HM III implantation. Her preoperative complete blood count, basic metabolic panel, coagulation profile, and hypercoagulability workup were unremarkable.

**Figure 1:**
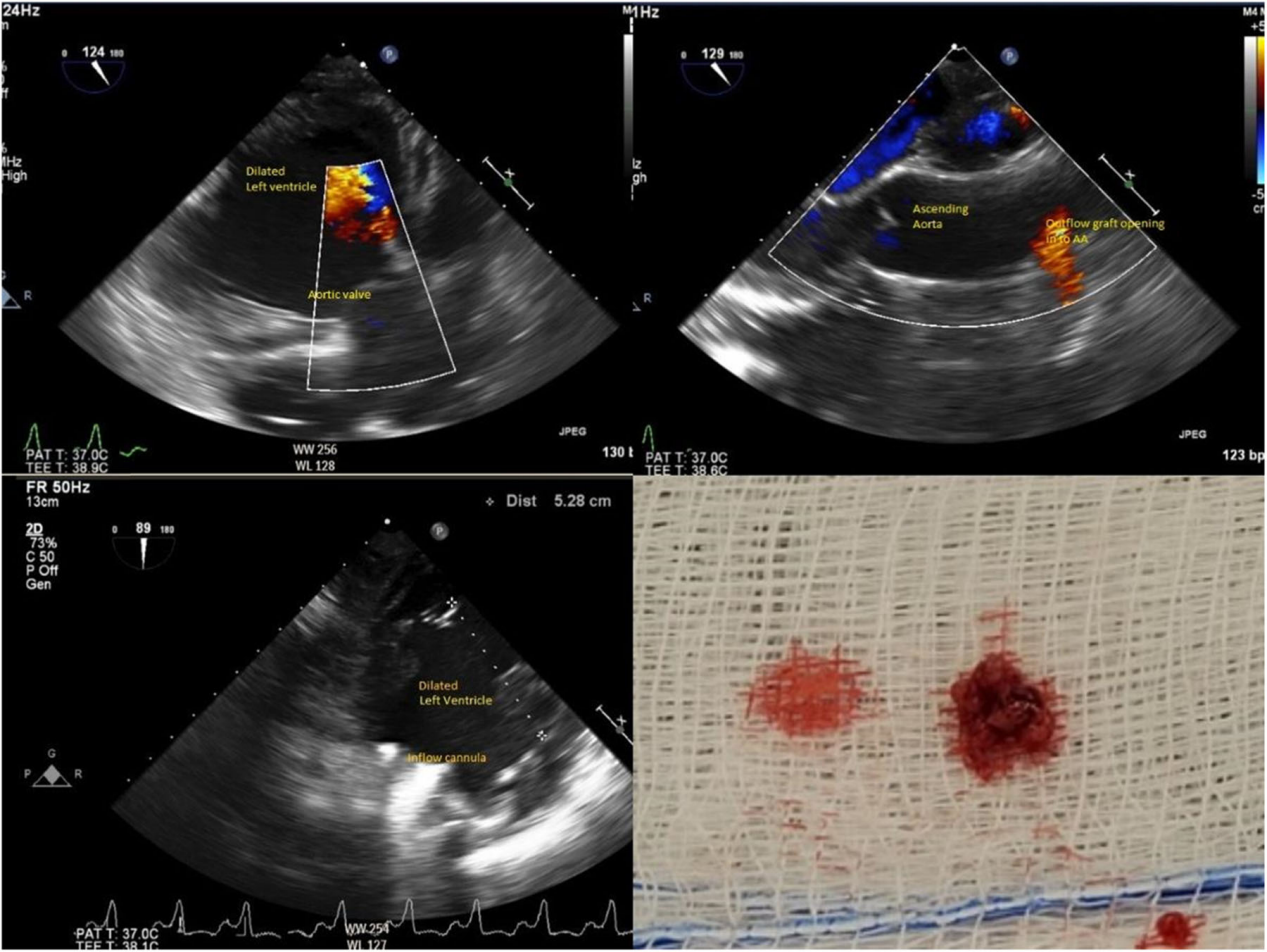
Intraoperative TEE images and the clot evacuated from the HM III (do we have VAD speeds) describe the images after naming them A−D. HM III, HeartMate III; TEE, transesophageal echocardiogram; VAD, ventricular assist device.

Perioperatively, there were no issues with anesthesia induction, maintenance, initiation, and weaning from cardiopulmonary bypass (CPB). One hour after the separation of the patient from CPB, a significant drop in ventricular assist device (VAD) flows and pulsatility index associated with an increase in power was noticed. Although the intraoperative transesophageal echocardiogram (TEE) did not reveal any obvious clot, The suspicion for PT was high due to left ventricle dimensions not decreasing with increasing the VAD speed (RAMP test). Moreover, the typical arterial waveform deflection associated with LVAD every 3−4 beats, as shown in Table 1 was missing. Further, there was an increase in the inotropic requirement for maintaining cardiac output and hemodynamics. Based on the above events, our team diagnosed acute intraoperative PT. The HM III was explanted; on direct inspection, the pump showed multiple small thrombi or fibrin‐like vegetation inside the inflow cannula and pump without any signs of external compression, kinking, and structural damage (Figure 1). The patient was placed back on CPB, and a new LVAD pump HM III was reimplanted, transferred to ICU with no issues. Post protamine point of care, thromboelastography was within normal limits. The explanted HM III device did not reveal any defects on further analysis by the manufacturer's.

**Table 1:** Normal arterial waveform and in patients with HeartMate III

Normal arterial waveform	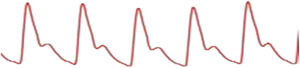
Arterial waveform in HeartMate III	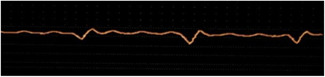

## 3 DISCUSSION

PT is a serious complication in patients receiving LVAD that often requires emergency thrombolysis and or surgery for pump replacement or can lead to death.^5^, ^6^ There have been reports of PT in HM III in the early and late postoperative period but none in the intraoperative period. In our case, we identified multiple small thrombi or fibrin‐like vegetation in the inflow cannula and the pump without any signs of kinking or compression of the inflow or outflow graft. The precise reason for acute intraoperative PT is unclear. We presume that the culprits can include any of the following, embolization of unidentified cardiac clots, low VAD flows, bleeding in the suture lines at the tissue graft interface, or scarred heart tissue from previous LVAD implantation serving as a nidus.^6^, ^7^

The PT diagnosis is based on insidious elevations in serum lactate dehydrogenase level (2.5−3 times more than the normal reference level), combined with clinical signs of hemolysis, CHF, and changes of pump parameters such as sustained power elevation more than 10 or 2 watts from the baseline.^7^, ^8^ However, diagnosing PT is challenging, especially in the intraoperative period, with limitations in doing imaging studies, lack of familiarity with intraoperative PT, and time sensitiveness. Available options intraoperatively to evaluate the integrity of the LVAD inflow and outflow graft include direct surgical inspection, changes in the pump parameters, TEE (Table 2), and the novel VAD endoscopy.^7^, ^9^

**Table 2:** Clinical signs, changes in pump parameters, and TEE findings associated with pump thrombosis

Clinical signs	Pump parameters	TEE
1. Cardiogenic shock 2. Absence of characteristic arterial waveform 3. Fatal arrhythmias 4. Pulmonary edema 5. Hemodynamic instability 6. Hypotension	1. Low power with low pulsatility index and steady pump speed: inflow/outflow obstruction, LV failure, dysrhythmia, hypertension 2. High power with low pulsatility index fluctuating pump speed: pump thrombosis, vasodilation, hypotension, initial response to exercise	1. Visible thrombus and or vegetation in the cardiac chambers or valvular structures 2. Right‐shift of the interventricular septum and left ventricle enlargement 3. Aortic valve opening with every beat (9−10/10 beats) 4. Blunted flow through both cannulas (pulse wave/color wave doppler) 5. RAMP studies (lack of LV dimensions change with increase in pump support/RPM)

Abbreviations: LV, left ventricular; TEE, transesophageal echocardiogram.

**Figure 2:**
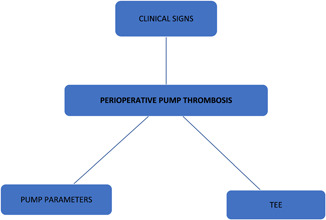
Diagnosis and management of pump thrombosis. TEE, transesophageal echocardiogram.

In our case, the TEE did not reveal any obvious clot or vegetations in the cardiac valves and chambers, both pre‐ and postimplantation of LVAD. We had a failed RAMP echo study.^10^ The RAMP echo study involves measuring echocardiographic parameters such as aortic valve opening time, left ventricle end‐diastolic diameters, deceleration time of the mitral inflow, and their changes between the lowest and highest pump speed settings.^10^, ^11^ Intraoperative TEE and RAMP echo studies can help in diagnosing the device malfunction associated with PT (Table 2). Figure 2 demonstrates the algorithm of the diagnosis and management of PT.

The management of PT depends on the type of occlusion, hemodynamic status, level of hemolysis, thromboembolic events, and end‐organ dysfunction. Management includes anticoagulation, thrombolysis, surgery for pump replacement, or heart transplant. However, the management of intraoperative PT is unique and requires immediate attention. Surgical pump replacement and or thrombectomy may be required to prevent acute hemodynamic collapse and thromboembolic sequelae.^12^ The use of anticoagulation, pharmacological thrombolysis is determined on a case‐by‐case analysis after weighing the risk and benefit of surgical bleeding status. Appropriate positioning of VAD pump, adequate VAD flows, early intervention can avoid PT and thromboembolic sequelae.^8^

To conclude, PT is uncommon with newer generation LVAD, especially in the intraoperative period, but it can occur. Diagnosing PT in the intraoperative period also poses significant challenges due to inherent limitations with the imaging, urgency, and lack of familiarity. Surgical thrombectomy and/or pump replacement are feasible options for intraoperative PT.
